# Miniaturized Sulfite-Based Gold Bath for Controlled Electroplating of Zone Plate Nanostructures

**DOI:** 10.3390/mi13030452

**Published:** 2022-03-17

**Authors:** Hanna Ohlin, Thomas Frisk, Mattias Åstrand, Ulrich Vogt

**Affiliations:** KTH Royal Institute of Technology, Department of Applied Physics, Biomedical and X-ray Physics, Albanova University Center, 106 91 Stockholm, Sweden; tfrisk@kth.se (T.F.); maastra@kth.se (M.Å.); uvogt@kth.se (U.V.)

**Keywords:** X-ray diffractive optics, zone plate, gold electroplating

## Abstract

X-ray zone plates made from gold are common optical components used in X-ray imaging experiments. These nanostructures are normally fabricated using a combination of electron-beam lithography and gold electroplating with cyanide gold baths. In this study, we present a gold electroplating process in a miniaturized gold-suplphite bath. The miniaturization is enabled by on-chip reference plating areas with well defined sizes, offering a reliable way to control the height of the structures by carefully choosing the plating time at a given current density in accordance with a calibration curve. Fabricated gold zone plates were successfully used in X-ray imaging experiments with synchrotron radiation. Although gold electroplating of nanostructures is a well-established method, details about the actual process are often missing in the literature. Therefore, we think that our detailed descriptions and explanations will be helpful for other researchers that would like to fabricate similar structures.

## 1. Introduction

Gold electroplating is a frequently used technique for the fabrication of gold micro- and nanostructures [1,2,3,4]. An important application area is X-ray imaging and X-ray microscopy, where different gold structures are used as optical elements, e.g., test patterns, gratings or zone plates [5,6]. Typically, these structures are fabricated by depositing a polymer resist on top of a substrate covered with a thin metal plating base, followed by either optical or electron-beam lithography. After development, the resist mold is filled with gold by electroplating, and removing the mold yields the final optical device.

Traditionally, cyanide-based gold plating baths have been used since they are very stable and reliable [2,5,7]. These baths are, however, highly toxic, utilizing cyanide gold complexes in combination with thallium- or arsenic-based brighteners. There are some alternatives like sulphite-based gold baths which often operate at higher pH. This might be problematic for certain resist systems, and exhibit much lower stability in such environments [4,8,9,10]. Moreover, standard plating bath setups are often quite big, utilising a large amount of chemicals [11,12]. From this point of view it would be desirable to use a less-toxic alternative and to reduce the bath volume as much as possible. The latter might be especially important for gold sulphite bath formulations, where the reuse of the liquid for consecutive plating experiments might be limited due to stability issues.

Electroplating for the fabrication of gold nanostructured features is very common, but ingoing explanations about the process or even which plating solution was used are often missing in the literature [1,6,11]. Factors such as current density, plating time and area will have an influence on the final outcome and are crucial for somebody else that wants to reproduce similar results.

In this paper, we describe a simple and low-toxicity approach for the fabrication of gold zone plate nanostructures. We use a sulphite gold bath with sub-10 mL volume and show how the gold electroplating process of zone plate nanostructures can be controlled with a direct current scheme in a miniaturized environment. Our approach is based on the use of a well defined on-chip reference plating area, which grants a way of controlling the plating height as a function of plating time at appropriate current densities. This is accomplished through measuring of calibration curves with data from several samples at the same current density, but different times. Devices fabricated through this method have been tested at synchrotron facilities, and the efficiency of such a device is presented together with a confirmation of its focusing performance.

## 2. Materials and Methods

### 2.1. Sample Design and Preparation

**Figure 1:**
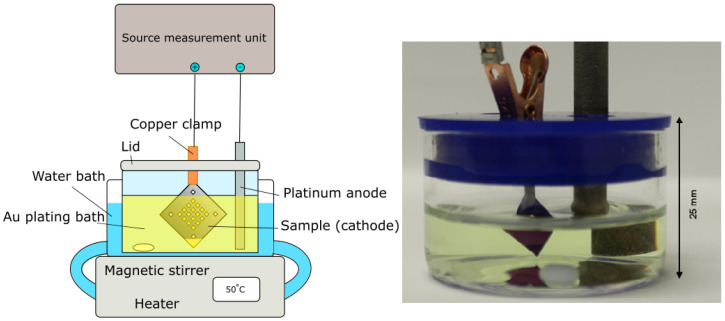
Picture of the experimental setup, indicating the size of the miniaturized bath, as well as a schematic of the full setup.

Samples for the study consisted of silicon nitride membrane chips (Silson, membrane thickness 100 nm and 200 nm, frame thickness 200 μm, pre-diced 7.5 × 7.5 mm${}^{2}$). Two types of chip layouts were used, one with in total 20 membrane windows and one with one central window. On the windows, different zone plate patterns (diameter 50 μm with outermost zone width 50 nm, and diameter 60 μm with outermost zone width 60 nm) were written with varying doses by e-beam lithography. Furthermore, two triangular fields were added to the chip designs. These act as the contact pad, kept above the plating bath liquid surface, and the reference plating area, which is submerged in the plating liquid at the at the opposing corner, for the electroplating step. This is shown in Figure 1. The design with nanostructures and reference plating areas on the same chip enables a miniaturization of the electroplating experiment which requires only a minimum amount of plating solution. At the same time, the area of the reference plating area is many times larger than the zone plates and will determine the plating speed for a given current density (see also Section 2.4).

All chips underwent an initial cleaning step with a reactive ion etch (RIE) oxygen plasma (15 s). This was followed up by a metallization step, applying a sticking layer of 10 nm Cr and a seeding layer of 5 nm Au through e-beam evaporation. Post-metallization, a second RIE oxygen plasma clean was performed.

### 2.2. Electron-Beam Lithography

Adhesion promoter (AR 300-80, Allresist GmbH, Strausburg, Germany) and electron-beam resist (CSAR 6200.13, Allresist GmbH, Strausburg, Germany) were applied through spin-coating at 3200 rpm for 1 min each. Spin parameters were selected to yield an approximate resist layer thickness of 450 nm. Adhesion promoter and resist were baked at 180 ${}^{\circ }$C for 2 and 3 min, respectively. Patterning was done with a Raith VOYAGER 50 kV electron-beam lithography system, and exposure of the zone plate nanostructures was made with an approximate starting dose of 210 μC/$cm^{2}$, dose scaling by decreasing feature size. Reference plating areas and contact pads were exposed with a lower dose. For zone plates, a low current 30 μm aperture (LC30) column mode was used, and for the utility structures a high current 70 μm aperture (HC70) mode was used in order to speed up writing. Chips were then developed in amyl acetate (AR 600-546, All-resist) for 60 s. The development process was stopped by submerging samples in IPA for 10 s, rinsed with pentane and left to air-dry. The patterning process was concluded with a brief RIE O${}_{2}$ plasma cleaning step to remove resist residuals at the bottom of the pattern (15 s).

### 2.3. Electroplating

A schematic of the plating setup can be seen in Figure 1. The plating bath consists of two components: the gold plating solution containing ammonium gold sulphite (AuH${}_{4}$NO${}_{3}$S) and an antimony (Sb) brightener solution based on potassium antimony(III) tartrate hydrate (C${}_{8}$H${}_{4}$K${}_{2}$O${}_{12}$Sb${}_{2}\;\;\cdot \;\;x$H${}_{2}$O). The exact composition of the solutions (Schütz Dental, Rosbach, Germany, EL-form S gold bath and El-form brightener) are proprietary, but according to the manufacturer the bath operates close to pH neutral and at an optimum temperature of 50 ${}^{\circ }$C. Optimum current density is not stated but a few mA/cm${}^{2}$ are common for similar solutions. The bath is originally designed for dental applications, and has been used previously in nanofabrication of gold nanotubes [1].

The bath was mixed by adding 7.8 mL of the electroplating solution and 0.75 mL of the Sb brightener to a 40 mL crystallisation dish together with a small stirring magnet, which was then placed into a water bath heated to 50 ${}^{\circ }$C. The dish had previously been cleaned with aqua regia. Before each plating a pH measurement (Eutech 3000 pH-meter) was conducted to ensure similar reaction conditions at pH 7.5 at 50 ${}^{\circ }$C. Samples were attached to a copper clip (Mueller, RS Components, Corby, UK) at the corner contact pad, and lowered into the plating bath together with a platinum-coated titanium anode. Special care was taken so that the clamp was not in contact with the plating solution. A constant current (DC) approach was chosen, and set currents and resulting voltages were controlled and measured through a source-measurement unit (SMU, Agilent U2722A, Santa Clara, CA, USA) with an in-house-written LabVIEW (NI, Austin, TX, USA) software. Post-reaction, the samples were removed from the copper clamp and briefly rinsed in de-ionized water before being left to air-dry. Finally, the resist mold was removed using reactive ion etch (Oxford Instruments Plasmalab, 20 sccm O${}_{2}$, 250 W ICP RF, 20 min, Abingdon-on-Thames, UK).

### 2.4. Calculating the Expected Plating Height

For our experiments, three different current densities were chosen, 0.9 mA/cm${}^{2}$, 1.8 mA/cm${}^{2}$ and 3.7 mA/cm${}^{2}$, and the outcome was studied at different plating times using a DC current.

To calculate the current that needs to be applied, we are dependent on precisely knowing the designated area to be plated. The total surface area of exposed nanostructures is, however, not guaranteed to be precisely defined in each experiment. Exposure errors due to dose variations or defocus are likely to occur. To amend this, the nanostructures are electroplated together with the much larger on-chip reference plating area, so that the surface area of the nanostructures becomes nearly negligible for current density calculations. This area is big in comparison to the nanostructures, and of a simple shape with a known area that is easy to expose with a guaranteed outcome. As an example, the total area to be plated for a given experiment was 1.6 × 10${}^{-6}$ m${}^{2}$, out of which only 0.14 × 10${}^{-6}$ m${}^{2}$ was the area of the zone plates (in this case 144 zone plates with 50 μm diameter each). With the area and the current, the applied current density during the plating process can be derived.

Theoretically, for DC current plating with 100% current efficiency and defined current density, the plating height *h* can be calculated as a function of the reaction time *t* with the following equation:

(1) $$h\left(t\right)=\frac{J\;\cdot \;m_{\mathrm{Au}}}{N_{A}\;\cdot \;e^{-}\;\cdot \;\rho_{\mathrm{Au}}}\;\cdot \;t$$

where *J* denotes the magnitude of the current density, $m_{\mathrm{Au}}$ the molar mass of gold, $N_{A}$ Avogadro’s number, $e^{-}$ the elementary charge, $\rho_{\mathrm{Au}}$ the density of gold, and *t* the plating time. Here we assume that the current density is constant over the whole chip.

The equation is based on volume calculations of the amount of gold atoms needed to fill the printed structures. As the gold sulphite complex yields Au${}^{+}$ ions in an oxidised state, each charge transferred will attribute to one deposited atom on the surface. As the current densities are known, the plating height *h* might be calculated.

### 2.5. Analysis of Fabricated Gold Zone Plate Nanostructures

Scanning electron microscope (SEM) images of plated gold structures were obtained using a FEI Nova 200 SEM at 10 kV. Height measurements of plated structures were done utilising profilometry (KLA Tencor P-15, Milipitas, CA, USA) with 0.5 mg stylus force. The zone plates were also used in X-ray imaging experiments at the NanoMAX beamline [13] at the MAX IV synchrotron radiation facility in Lund, Sweden, including measurements of the diffraction efficiency.

## 3. Results and Discussion

### 3.1. Plating Height as a Function of Current Density

**Figure 2:**
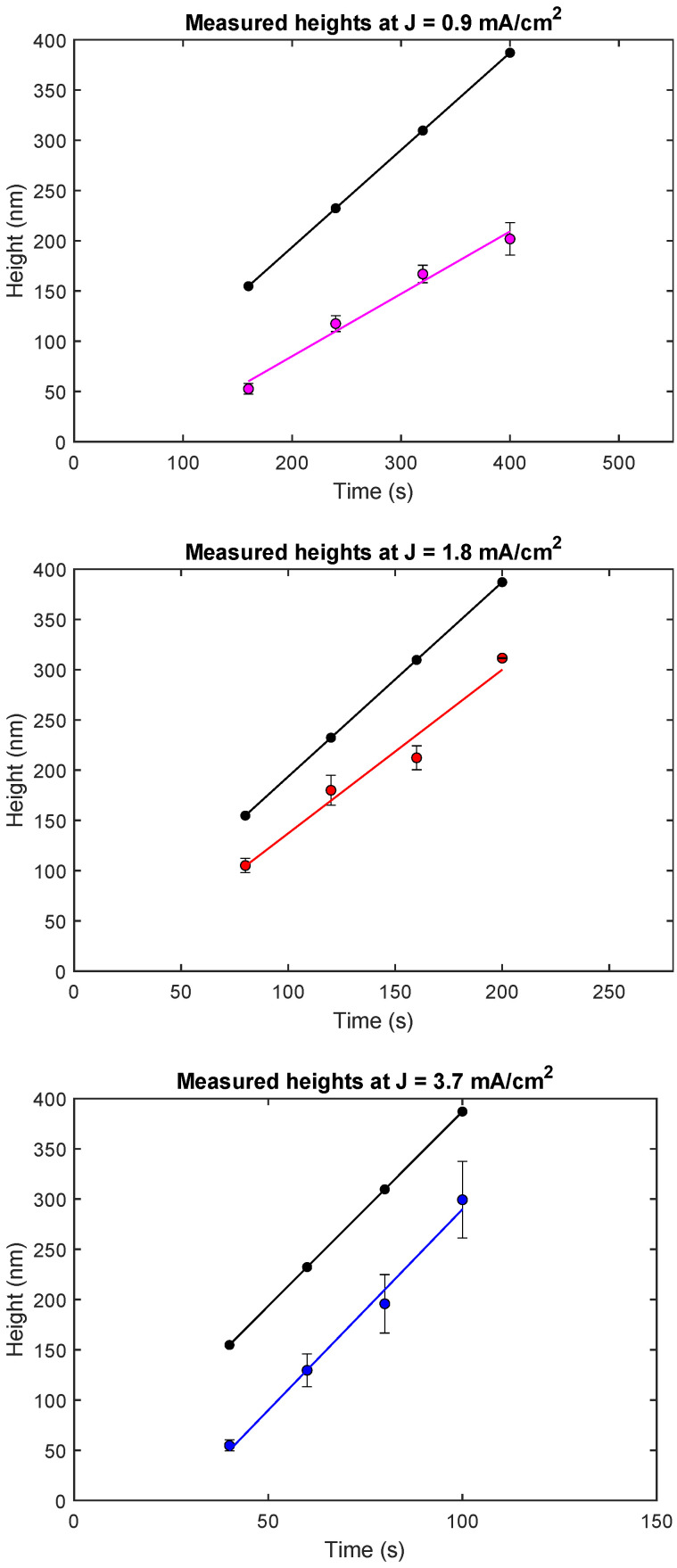
Current density plots, with the theoretically calculated height marked as the topmost black curve in each plot. Experimental values are shown in coloured dots, together with the linear trend between the samples in each series. Each point along the lines consist of an aggregate of average values of several measurements on the same sample in every series. 2σ error bars are shown.

Figure 2 shows the results from three current density experiment series, where the plating height was measured as a function of reaction time (for the 50 μm diameter zone plates). Each plot contains two different sets of data points. The theoretically calculated values according to Equation (1) are shown in black. The coloured points mark the experimental values. Each line is a linear fit to the respective data set. It should be noted how the time values vary between each plot. This is because the experiments were designed to reach similar plating heights. As can be seen in the plots, there is a clear linear trend between reaction times and plating height. The linearity suggests that the plating height can indeed be controlled by plating time. This also proves that the experiments are reproducible, and by utilizing the data as a calibration curve for a certain current density, resulting heights can be reliably estimated.

Although all three experimental series display a linear behaviour, there are discrepancies between the experimental and the theoretical values. All measured experimental values lie below the calculated series to a varying extent, with a few differences between the current densities. Additionally, the slopes of the linear fits are in the first two cases not the same for different current densities. These results show that Equation (1) can only be used to calculate a first rough estimate of the plating height. Hence, calibration experiments are needed in order to be able to predict plating height as a function of plating time for each current density. Once calibration curves are obtained, we find that plating experiments can be reliably controlled and that they are reproducible.

**Figure 3:**
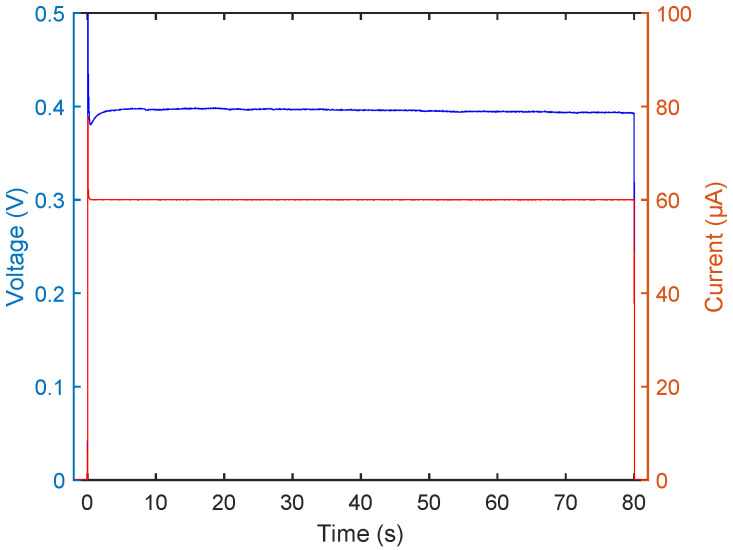
Current and voltage plotted for a standard plating experiment. Current plotted in red, voltage plotted in blue.

To further understand the experiment, curves for current and resulting voltage from the plating reaction can be studied, as shown in Figure 3.

The current curve, shown in red, is, after an initial spike caused by the slow response of the SMU regulating loop, stable at the set value. A similar behaviour can be seen for the voltage, shown in blue, which spikes initially prior to swinging back to a stable value. In this fashion, the success of the experiment can be assessed in real time, which provides a reliable way to follow the plating process. A dip in voltage indicates a fault in the experimental setup, such as short-circuiting or other components than the sample alone contacting the plating bath, in which case the plating reaction does not proceed in accordance with the calibration curve.

### 3.2. Imaging and Characterization of Fabricated Zone Plate Nanostructures

**Figure 4:**
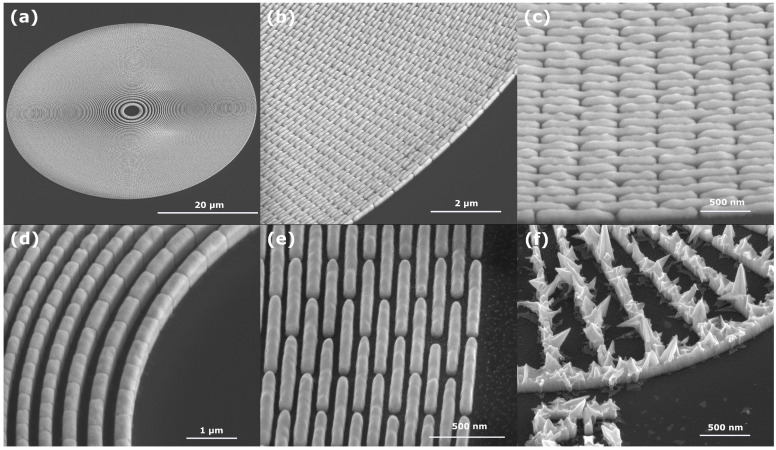
SEM micrographs of electroplated structures. (**a**) Overview of a zone plate with 50 nm outermost zone width. (**b**,**c**) Details of the outermost 50 nm zones. Plating height is approximately 280 nm. (**d**) Innermost zones of a 60 nm zone plate, plating height nearly 450 nm. (**e**) 60 nm outermost zones. (**f**) Siemens star structure electroplated without the Sb-brightener. Distinct growth of crystalline structures throughout the pattern can be seen.

In Figure 4, several SEM micrographs are shown of both 50 and 60 nm feature size zone plates, shown depicting a full zone plate (a) and details of the outermost zones in two different magnifications (b, c, e) as well as an overview of the innermost zones (d). A few negligible imperfections towards the outermost zones can be seen in the case of the 50 nm structures. This can be attributed to slight errors in the process that defines the lithography pattern. As shown in the pictures for both the 50 and 60 nm structures, individual features are well defined and uniformly plated with no overplating, misshaped structures or other faults.

**Figure 5:**
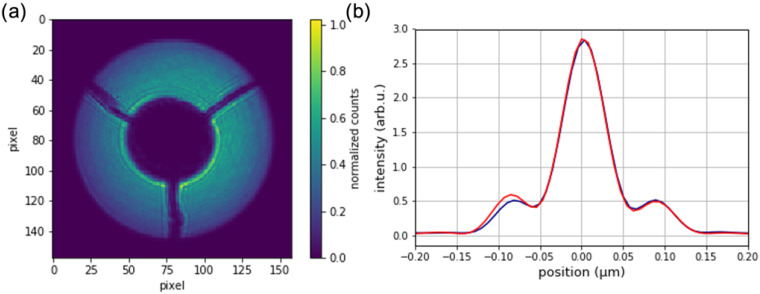
(**a**) First order diffraction cone of a fabricated zone plate with 60 nm outermost zone width. The round disk with three support bars in the center is the central stop. (**b**) Focal spot profile in horizontal (blue) and vertical (red) direction reconstructed by ptychography. The full width at half maximum size is 60 nm × 60 nm.

Fabricated zone plates were successfully used in imaging experiments with synchrotron radiation. Figure 5a shows the first order diffraction cone of a 60 nm outermost zone width, obtained at the NanoMAX beamline at the MAX IV laboratory. The zone plate was coherently illuminated with an X-ray beam of 8 keV energy. A gold central stop was placed in front of the optic, and an order-sorting aperture was used to single out the first diffraction order. The image was obtained on an Eiger2 4M detector about 4 m behind the zone plate. The intensity map can be interpreted as the local diffraction efficiency, which is not completely homogeneous over the whole optic. The reason is a slightly lower plating height for smaller structures towards the outer parts of the zone plate. Nevertheless, the optic works as expected and can focus the X-ray beam to the diffraction limit, as seen in Figure 5b. The data was obtained by ptychographic imaging of a test object [14]. The efficiency of the zone plate was measured to be 3.3%. To obtain this number, the total counts in the first diffraction order were divided with the total counts of the direct beam (without central stop and optics), transmitted through the order-sorting aperture and corrected for different diameters.

### 3.3. Plating Bath and Operation

The bath used for this study is originally developed for dental applications. Nevertheless, it worked very well for plating of nanostructures. A possible advantage compared to other commercial sulphite baths dedicated for micro- and nanofabrication is the fact that it operates at lower pH close to 7, which might be important for the stability of some resists. After using plating mixtures once for the processing of a low number of samples, the liquids were stored in closed vials at room temperature to observe long-term stability. Whilst it is well know that a sulphite gold plating bath has a considerably lower stability than a cyanide gold bath [9], it should be noted that the stability of the plating solution we used is still very good, especially prior mixing the final bath. From our experience, usability of the base solution extends past suggested shelf life without any issues. We think that storing the gold sulphite and brightener solutions in different containers might increase the real shelf life considerably, and it is only after the two components are mixed that the resulting plating solution shows tendencies to precipitate, which normally occurs after 6–8 weeks. After this point, macroscopic precipitation of gold was observed in used baths. We assume that this might happen independently of the bath volume, highlighting another possible advantage of using only a small-volume setup.

The advantage of building a small-volume setup is that the amount of chemicals needed is severely reduced. This is enabled by the on-chip electrode design in combination with utilising small-sized chips over larger wafers, which would have required a plating bath of bigger volume. For the relevant applications in this case, the amount of gold in the solution is sufficient to plate a significant amount of chips, and the same plating solution has been used for consecutive plating of samples without issues. We found that the integration of a stirrer matters greatly and is, besides the chip size, a limiting factor for how much one can miniaturize the bath. Without a stirrer, the plating of structures higher up in the bath was considerably lower compared to structures closer to the bottom, although the distance between them was only a few millimeters.

We observe that plating of nanostructures with good quality was possible with a DC current approach. This is in contrast to work by Zhu et al. [5] which found pulse plating necessary for a smooth filling of the mold (although at 30 nm outermost zone instead of 50 nm). We think that DC plating is preferable if possible, since it eliminates two parameters, frequency and duty cycle, and simplifies the process. From our understanding of the literature, pulse plating for gold was mainly studied as an alternative to operate a bath without the addition of brighteners and still obtaining dense films [15]. To further investigate this, a plating attempt was made without the addition of the brightener solution. In Figure 4f, a Siemens star structure electroplated in this way is shown, which yields a spiky crystalline growth. This suggests that in order to obtain a smooth filling of the nanostructures with DC plating, the brightener solution is an absolutely necessary addition to the bath [2,16,17]. With this approach, good and dense filling of the structures can be obtained. It remains to be investigated if pulse or pulse reverse plating schemes can be beneficial for a more uniform filling of structures with different sizes [18].

## 4. Conclusions

In conclusion, we present a detailed description of the start-to-finish gold electroplating process of zone plate nanostructures with outermost zone widths of 50 nm and 60 nm. These have been fabricated in a reproducible way utilising a sulphite gold electroplating bath with antimony brightener and a direct current plating scheme, which has been studied and described in detail. By current density calculations and the use of an on-chip reference plating area, controlling plating height by selecting the relevant plating time is facilitated via calibration curves for relevant current densities. The plating bath is miniaturized, only utilizing a minute amount of plating chemicals for each sample, and has yielded structures of consistent quality down to a feature size of 50 nm. Devices fabricated by this method have been used for X-ray imaging experiments with synchrotron radiation. We think that our results and detailed explanations are valuable information for other researches that want to start producing gold nanostructures by electroplating.
